# A new, ‘hip’ way to breathe

**DOI:** 10.7554/eLife.70947

**Published:** 2021-07-06

**Authors:** Marc R Spencer

**Affiliations:** Department of Anatomy and Cell Biology, The George Washington Washington UniversityWashington, DCUnited States

**Keywords:** ornithodira, dinosauria, ornithischia, dinosaurs, Other

## Abstract

Ornithischians, one of the three major groups of dinosaurs, developed a unique mechanism to ensure airflow in the lungs.

**Related research article** Radermacher VJ, Fernandez V, Schachner ER, Butler RJ, Bordy EM, Hudgins MN, de Klerk WJ, Chapelle KEJ, Choiniere JN. 2021. A new *Heterodontosaurus* specimen elucidates the unique ventilatory macroevolution of ornithischian dinosaurs. *eLife* **10**:e66036. doi: 10.7554/eLife.66036

Breathe in… and out. As your chest rises and falls, the diaphragm and several lesser-known muscles create the reassuring bellows-like motion that allows air to fill and leave the lungs (Perry et al., 2010). All living mammals and many extinct relatives share the same respiratory muscles and ‘ventilation’ technique, but this is not the only way to breathe.

For example, archosaurs, a large group of reptiles that included all dinosaurs as well as the ancestors of crocodylians, used ‘cuirassal breathing’ instead (Carrier and Farmer, 2000). They had oddly shaped bones known as gastralia in their abdominal wall, which assisted in ventilation by helping to connect the rib cage to the muscles and bones in the pelvis. With time, however, the architecture of the pelvis changed, and structures other than gastralia took over to help breathing in crocodylians and many dinosaurs, including birds (Baumel et al., 1990; Farmer and Carrier, 2000). For example, in birds, the pelvis rotated and a new, complex ventilation system emerged; in crocodylians, a ‘hepatic piston’ developed, whereby a muscle anchored to pubic bones in the hip pulls back the liver to create a motion that draws in air into the lung.

Amongst dinosaurs, two groups (one extinct, the other which gave rise to birds) feature early species with gastralia, only to lose these bones in favor of other ventilatory mechanisms later in evolution (Claessens, 2004). On the other hand, gastralia had never been found in species belonging to the now-extinct third dinosaur group Ornithischia, which later on included species such as *Triceratops* and *Stegosaurus*. Now, in eLife, Viktor Radermacher and colleagues report having found, for the first time, gastralia in Ornithischia (Radermacher et al., 2021). The team, which is based in institutions in Canada, South Africa, the United Kingdom, France and the United States, spotted the bones in *Heterodontosaurus*, one of the oldest-known ornithischians.

Beyond the unique presence of these bones, this new *Heterodontosaurus* specimen from South Africa also displayed features of other ornithischians, such as sternal plates. These peculiar bones of the chest wall may have facilitated cuirassal breathing in some early archosaurs – including, as Radermacher et al. now reveal, in early species of ornithischians.

*Heterodontosaurus* possesses a small projection on the pubis—one of the three bones of the hip—that points toward the head. This ‘anterior pubic process’ grew longer as Ornithischia evolved during the Mesozoic Era, while the main portion of the pubis decreased (Figure 1). The elongation remained unchanged even though the body plan of ornithischians became altered, and certain later species switched from walking on two legs (like *Heterodontosaurus*) to moving on four. This suggests that the anterior pubic process was part of a potentially new breathing apparatus (Brett-Surman, 1989; Norman, 2021).

**Figure 1.**
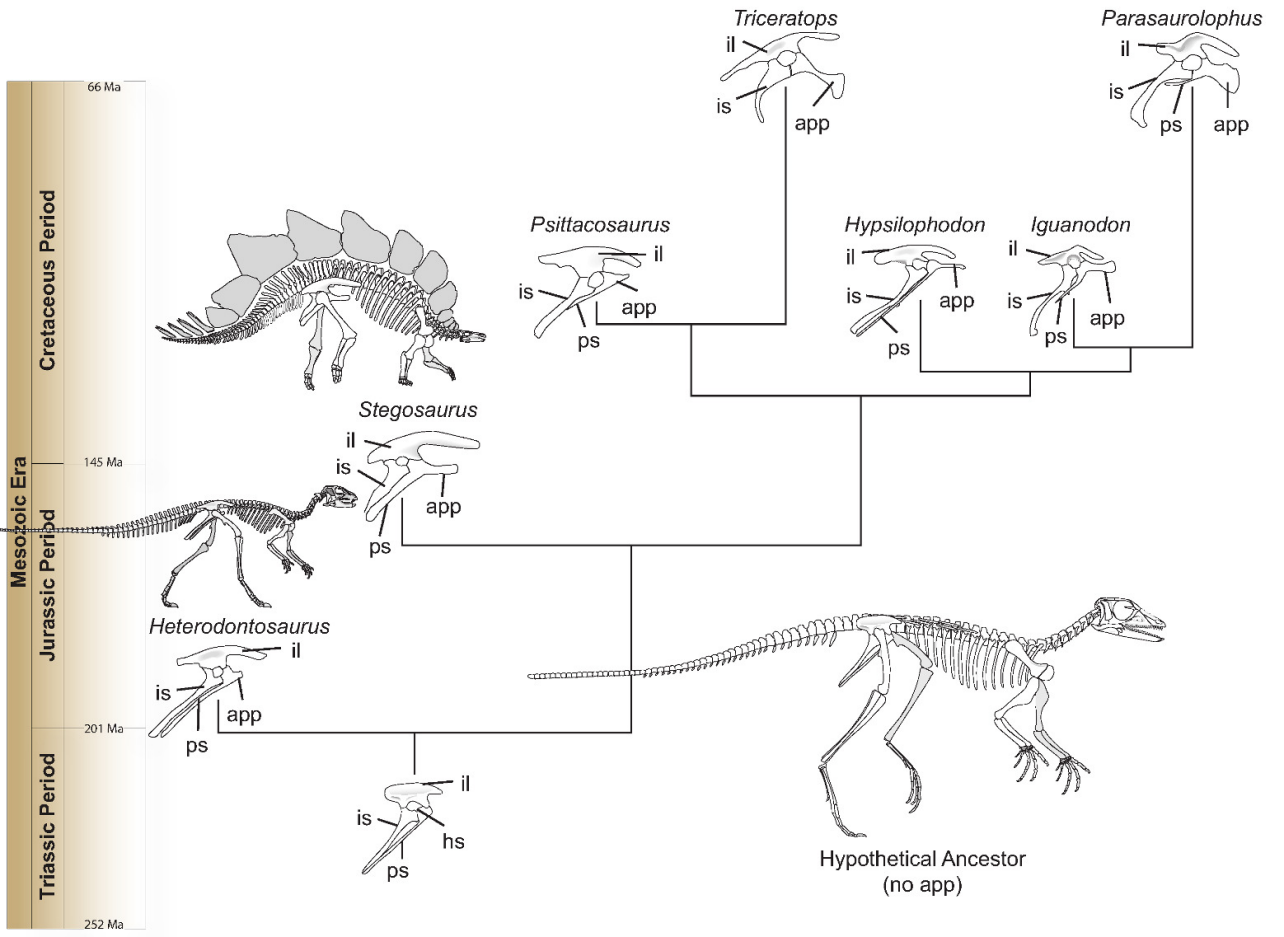
Hip evolution in ornithischians. A simplified phylogenetic tree illustrating the evolution of the various components of the ornithischian hip from the Triassic Period (bottom) to the Cretaceous Period (top; **app**: anterior pubic process; **hs**: hip socket or acetabulum; **il**: ilium; **is**: ischium; **ps**: pubic shaft). The hypothetical ornithischian ancestor (full skeleton, bottom right) lacks an anterior pubic process, which is a projection of the pubis bone pointing towards the head. As Radermacher et al. demonstrate, this structure was present in *Heterodontosaurus*, an early ornithischian which walked on two legs. During evolution, the process grew bigger, and is thought to have helped anchor a muscle responsible for pelvic bellows, a new breathing mechanism which could have persisted even when ornithischians started to move on four legs (like, for example, *Stegosaurus*). All hips are oriented in the same direction and are not to scale. Estimated dates given in millions of years (Ma) and taken from Cohen et al., 2013.

Image credit: Christopher Brochu created the skeletons of *Heterodontosaurus* and *Stegosaurus*.

Radermacher et al. then examined numerous specimens from other species that represented all of the major groups in Ornithischia. Following this thorough survey, the team suggests that gastralia became decoupled from the breathing process early in ornithischian evolution, as the anterior pubic process started to elongate in the hip (Figure 1). Instead, a new ventilatory mechanism could have been in place. In this model, the anterior pubic process could have served as an anchor for an hypothetisized ‘puboperitoneal muscle’ that grew more robust and important throughout the evolution of ornithischians. As it contracted, this muscle would have stretched the posterior portions of the lungs to expand the body cavity during respiration, creating so-called ‘pelvic bellows’. This breathing apparatus, though similar to the hepatic piston found in crocodylians, would have been truly unique amongst other dinosaurs (Schachner et al., 2014). In turn, the presence of the anterior pubic process diminished the need for additional structures to facilitate ventilation, and gastralia or other elements associated with cuirassal breathing (such as the sternal plates) shrank or disappeared.

This proposed pelvic bellows model will, no doubt, prompt more investigation, given that few have examined the potential ventilatory mechanisms in Ornithischia. In particular, future works on later groups of ornithischians should explore whether the shift to walking on four legs was, as Radermacher et al. suggest, dissociated from changes in the anterior pubic process.
